# Tension, depression, and culture among women in rural Bangladesh: Continuity and change

**DOI:** 10.21203/rs.3.rs-10010547/v1

**Published:** 2026-06-25

**Authors:** Lesley Jo Weaver, Fahmida Tofail, Alison Karasz

**Affiliations:** University of Oregon; International Centre for Diarrhoeal Disease Research; University of Massachusetts Chan Medical School

**Keywords:** women, Bangladesh, ethnography, common mental disorders, LMIC, rural

## Abstract

People’s experiences of psychological distress can provide insight into broader social contradictions and divisions in societies. In Bangladesh, an ethnographic record dating back to the 1920s presents an opportunity to explore how those contradictions and divisions have changed or stayed the same in 100 years. This article draws on interviews with a group of 60 women with likely depression in rural Bangladesh to a) explain their distress, its causes, and its possibilities for resolution; and b) compare those experiences to the ethnographic record. Social institutions reflected in the literature, like dowry, patrilocality, and mobility restriction, were still key features of life for women in the study and were significant sources of distress in their everyday lives. On top of these “classic” stressors, newer stressors also surfaced from women’s narratives, including debt to microlending programs and husbands’ addictions to pharmaceuticals. Women articulated an awareness of emerging opportunities for girls and women in rural Bangladesh that did not exist a generation ago, such as pursuing education, marrying later, having smaller families, and working in a job. They hoped for their children to benefit from these new opportunities, but their aspirations were often thwarted by ongoing poverty and patriarchal norms that limited their life chances.

## Introduction

People’s experiences of psychological distress provide insight into broader social contradictions, tensions, divisions, and processes of change in societies. In the now-classic *Illness Narratives*, psychiatrist Arthur Kleinman observed, “The illness experience is a key form through which social worlds are apprehended and given meaning, for it is through suffering that the moral and social order becomes visible in everyday life” (1988: 31). Beginning from the notion that people’s accounts of suffering are important sources of information about their social worlds, this article examines how women with likely depression in rural Bangladesh explain their distress, its causes, and its possibilities for resolution. Their narratives reveal how large-scale social and economic sources of marginalization manifest within their own lives, and provide insight into cultural change and continuity in this region over the last 100 years.

## Ethnography among women in rural India and Bangladesh: Patrilocality, Purdah, and Dowry

A body of ethnographic work dating back to the 1920s documents the challenging lives of women in rural northern India and Bangladesh, which functioned as a unified territory until the Partition of 1947. Prominent themes from this early work include *patrilocality, purdah*, and *dowry* and their influences on women’s lives. We briefly review each of these below.

First, early ethnographic work detailed how rural women’s lives were patterned by *patrilocality*—the practice of women moving into their husbands’ and in-laws’ household after marriage (Wiser & Wiser, 1930). In the 1920s, most village women were unable to access formal education, and girls were often married shortly after menarche, at which point they entered their husbands’ households as the most junior member. This post-marriage move was often a moment of “radical change” for a young woman (White, 1992: 96), and it reinforced gendered power dynamics within households. A new “wife, and any other women who are junior to [the husband], are ready to do his bidding with heads bowed and voices subdued,” wrote Wiser and Wiser in the 1930s (1965: 79). An ideal daughter-in-law “should accept what the household people say, whether they are right or wrong” (Wadley 1994:62). Young brides mainly worked in the home under the supervision of their mothers-in-law, which often resulted in bitter intergenerational conflict (a phenomenon well documented across South Asia into the present (Dube, 1997; White, 2017)). If a woman wished to leave an unhappy marital household, she could go back to her natal family for some time, but she might be compelled to leave children behind to ensure her eventual return (Wiser and Wiser 1965).

Second, the ethnographic record contains many references to *purdah* (women’s Islamic seclusion) and its impacts on women’s decision-making power and physical mobility outside the household (Jeffery, 1979; Liddle & Joshi, 1989; Mandelbaum, 1988; R. Nazneen, 1996; Vatuk, 1982). These sources describe purdah as a mechanism for regulating women’s sexuality and as an important public performance of both the family’s status and the family’s adherence to Muslim observance. To avoid the negative judgments of others, women’s social roles were generally circumscribed to domestic work and the birth and care of children (Jeffery, 1979; Wiser & Wiser, 1971). “The woman is inferior,” explained an older woman involved in Wadley’s work in the 1980s. “A woman can only work according to the regulations. She can never leave the regulations. If our women go out in the world or my daughters wander in the world, I may be the target of defamation, as well as my dead husband” (Wadley, 1994: 38). Purdah also resulted, more practically, in the construction of women as legal minors who could not own land, hold private assets, or make decisions without their husbands’ input (Mandelbaum, 1988).

Third, the ethnographic record discusses *dowry* as a key source of conflict in women’s marital homes. Dowry was (and still is) a widespread practice across South Asia, despite the fact that its abolition has been advocated since the 1970s (Liddle & Joshi, 1989; Mandelbaum, 1988). Lavish dowries were a status symbol and an indirect form of compensation to the in-laws to offset the bride’s support, and insufficient dowry could jeopardize a bride’s security within her marital household. The natal family’s financial obligation to the groom’s family often did not end at marriage, but might continue through “giving gifts to her and her marital family on the proper occasions, particularly in the early years of her marriage” (Mandelbaum 1988: 68). The high cost of dowries resulted in a situation where parents felt “a terrible pressure to get [daughters] off their hands at the lowest cost” and as early as possible (White, 1992: 102). In these ways, dowry perpetuated early marriage and young women’s vulnerability in their marital households.

Although regulations around patrilocality, purdah, and dowry were culturally normative, they might be relaxed among families of lower castes or minimal means, which often split into nuclear units due to a combination of financial and emotional pressures arising from land and resource scarcity (Lieten, 1992; Schendel, 1981). For some areas, “Fissioned households are the rule, particularly after the parents have passed away,” while joint family arrangements persisted primarily in households with large amounts of land where large families were needed for farm work (Lieten, 1992: 40). Women in poorer households might work as day laborers or house cleaners out of sheer economic necessity, even though this meant breaking purdah rules (Liddle & Joshi, 1989; Schendel, 1981; Wadley, 1994; Wiser & Wiser, 1930).

Twenty-first century ethnographic work documents changes in many aspects of women’s lives, while other practices and institutions persist (Chowdhury, 2018; Karim, 2022; Nazneen et al., 2011). Women in Bangladesh have been the focus of government and non-government social change projects aimed at improving their life circumstances since the 1970s (Islam & Sayeed, 2024; Nazneen et al., 2011). Consequently, women have new pathways to economic participation, such as microlending or work at international garment factories (Karim 2016, 2022). Girls’ access to free and subsidized primary and secondary education, women’s access low-cost reproductive healthcare, including birth control, and women’s overall life expectancy have increased dramatically (Wilce, 1998; World Bank, 2002; World Health Organization, 2025). Child marriage is now illegal, though it still occurs among up to half of women in Bangladesh (UNFPA-UNICEF, 2025).

Given these significant changes, we ask, to what extent might the lives of rural women remain influenced by structures like patrilocality, purdah, and dowry that were so prominent in the early- and mid-20th century ethnographies? And, how do women’s experiences of distress reflect or challenge these structural factors?

### Mental health, “tension,” and distress in South Asian women

Since its 1971 independence, Bangladesh has seen significant improvement in many public health indicators (Directorate General of Health Services, Bangladesh, 2025), but mental health has emerged as a challenge. The 2021 Global Burden of Disease study reported a 5.4 percent prevalence rate for depression across Bangladesh, roughly equivalent to the US’s 5.5 percent, and significantly higher than the 4.1 percent reported in 2015 (IHME, 2015, 2025). Particularly among rural groups, which comprise an estimated 60 percent of Bangladesh’s population (World Bank, 2018), access to basic healthcare remain deeply challenging (Kamal et al., 2016; Rahman et al., 2024; Zahangir et al., 2017). These estimates may, therefore, be artificially low.

“Tension” (the English word, which has been incorporated into many South Asian languages) is a common term for talking about distress in South Asia and diaspora populations, including in Bangladesh (Karasz, 2005; Weaver & Karasz, 2022; Wilce, 1998). Tension can refer to varied distress experiences ranging from small annoyances such as misplacing items, to serious and potentially debilitating distress. Tension often functions as a metaphorical link between external life stressors and internal thoughts and feeling; for example, tension can refer to the psychological state of being in tension, to specific worries, and even to causal events. The work on symptom comparison shows significant overlap between tension and depression (Karasz, 2005; Weaver, 2017), but unlike depression, tension is not generally characterized as a disease state. Instead, tension is described as a condition of mental strain that can lead to physical or mental health problems if it is severe or remains unresolved (Weaver & Karasz, 2022).

The present ethnographic study was embedded in a depression treatment trial. The overall goal of the ethnographic study was to learn, through discussions of tension, about the broader challenges women face in rural Bangladesh, and to develop insights about how their everyday life experiences contributed to their experience of depression treatment in the trial.

## Methods

### Setting

This study took place in Dinajpur, an agricultural district in northern Bangladesh. Dinajpur is part of Rangpur district, one of the poorest divisions in Bangladesh, with a very strong presence of national and international development organizations focused on women’s empowerment such as BRAC, World Vision, Caritas, and Grameen Bank. These organizations have been heavily involved in development projects, especially in agricultural skill development, health promotion, savings programs, and micro-lending for local women.

## Sample, eligibility, and larger study context

Sixty low-income women with probable depression living across 4 villages constituted the study sample. These women were recruited from a larger sample of 710 participants enrolled in a randomized controlled trial (RCT) testing two treatments for depression. The RCT included two treatment groups: one receiving Problem Management Plus (PM+), a brief, manualized therapy for depression supported by the World Health Organization (Dawson et al., 2015), and the other receiving both PM + and a poverty alleviation intervention modeled on the Graduation Program (Matin et al., 2008). The PM+ program teaches problem-solving strategies, emotion regulation, behavioral activation, and building social support and has been shown to reduce depressive symptoms in many global contexts (Cai et al., 2025). The Graduation Program includes asset transfer, financial education, savings, and gardening, and has been shown to improve financial security in a range of humanitarian contexts, including in Bangladesh (Hashemi & de Montesquiou, 2024). Women received a total of 10 integrated group sessions over six months.

All women in the RCT had screened positive for likely depression, based on a score of > = 10 on a Bengali version of the PHQ-9, a depressive symptom screening tool consisting of 9 items that has a possible score ranging from 0 to 27. Other inclusion criteria included poverty—defined for this study as monthly per-capita household income < 15,000 Bangladeshi takas (about $122 USD)—basic literacy, and fluency in Bengali.

## Data collection

Semi-structured interviews were conducted by 13 Bangladeshi research fellows under the supervision of one Bangladeshi senior researcher and two American researchers with extensive experience in community-based qualitative and ethnographic data collection and analysis. Interviews occurred in Bengali language and were conducted in a private location at participants’ homes. They lasted between 30–120 minutes. Questions addressed women’s life histories, everyday routines, social lives, sources of stress, hopes for the future, loneliness, cultural norms, and sources of support. Interviews were voice recorded, translated to English, and coded bilingually in both Bengali and English to identify common themes across the data set. All study procedures were pre-approved by the Institutional Review Boards of [BLINDED—includes one IRB in Bangladesh and one in the US].

### Analysis

Our coding procedure followed Braun and Clark’s “stepwise thematic analysis with a codebook” process to identify both semantic and latent themes (Braun & Clarke, 2006). Team members read through a portion of the transcribed interviews and participated in a group discussion to develop a preliminary codebook consisting of both *a priori* (deductive) codes derived from the interview guide, and inductive codes derived from reading and discussing interviews (Braun & Clarke, 2006). After coding five interviews and conducting informal inter-coder reliability checks, the group adjusted the codebook and our own coding procedures as necessary, then repeated this procedure until no new codes were found and coders reliably coded in a similar manner. Transcripts were back-coded with the final codebook as necessary.

After initial coding, we sorted codes into initial broad “buckets” of meaning around tension and its causes, which we further divided into sub-themes representing the most commonly-discussed specific causes of distress.

## Results

### Sample characteristics

Participants were on average 30 years old, majority Muslim, and married. All had been married before the age of 18. Over ninety percent had current outstanding loans, often more than one. They had an average of six and a half years of education, two children, and over half cohabitated with their in-laws in a joint family. See Table 1, below, for a full report of demographic characteristics.

As expected based on prior research and on the RCT implementation materials, the most common term women used to discuss distress was the English term *tension*. Also common were the Bengali terms *koṣṭo* and *pērēśāni*, both of which translate roughly as “troubles.” Only one of the 60 participants had heard of the Bengali term used for clinical depression, *biṣaṇṇatā*. Women described tension as consisting of both physical symptoms and emotional distress, in line with prior work on the symptoms and features of tension. One woman explained, “My heart races (*buk dhorfor kore*), one side of my chest constantly aches (*buker ekpash betha kore*). I don’t feel good (*bhalo lage na*), I feel restless (*osthir lage*). Many days go by like this—I can’t even sleep at night from all the stress. There’s so much tension in this household!” Consistent with other studies of distress in South Asia, tension was understood as the suffering experienced by both the body and the mind as a result of externally-imposed circumstances (Karasz, 2005; Roberts et al., 2020; Weaver, 2017; Weaver & Karasz, 2022). Tension was ubiquitous for many women in this resource-constrained setting. Smiling sadly, one woman explained, “Everyone has many worries. Does tension have a limit? It’s in everything. How will I build a house? How will I educate my children? How will I raise them to be good people? If I’m alive, what will I eat, what won’t I eat? If I live, I’ll have to worry about food, I’ll have to think about earning and spending. That’s also a tension.”

### Thematic results

**Figure 1:**
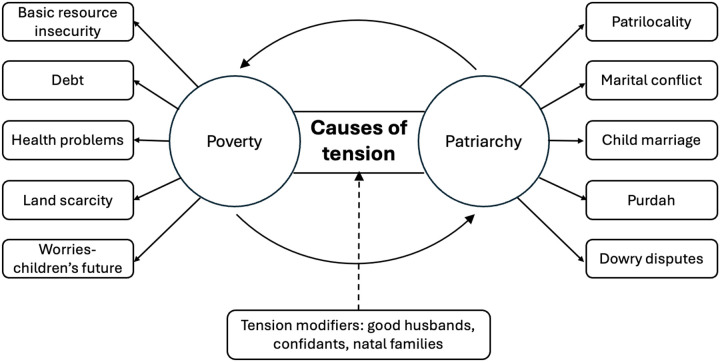
The most common causes and modifiers of tension among women in our study.

The main meta-theme we constructed was “causes of tension.” This comprised two major themes: poverty and patriarchy, which exist in relationship to one another and may be mutually reinforcing, as shown by the cyclical relationship in Fig. 1. The major themes of poverty and patriarchy each contained five sub-themes identifying the specific causes of tension women described most often. We also noted three major factors that could reduce tension in women’s lives: good husbands, confidants, and the support of natal families. These stressors and modifiers were not discrete features of the local context, but rather were parts of a mutually-reinforcing total experience that comprised women’s life circumstances. See Fig. 1, below.

### Theme 1: Poverty and its attendant circumstances

Without exception, the women in this study were living under conditions of extreme poverty, and this generated tension in their daily lives. Sub-themes included basic resource insecurity, debt, health problems, land scarcity, and worries about children’s future.

#### Basic resource insecurity

All families in the study were impoverished, based on inclusion criteria. All engaged in some combination small-scale agricultural production and day labor for subsistence, both of which fluctuated seasonally and depended on family members being well enough to work. “If there’s work, we buy rice and eat,” explained one participant, who said that sometimes the family goes entire days without food. “We don’t have our own paddy or rice stock to depend on. We do sharecropping on rented land, using money to cultivate.” Another woman explained, “What can I say about my worries? The main issue is survival. My family is growing bigger day by day. There is only one earning member. Food is a big concern—though I’ve married off one daughter, I still have five more to care for. Sometimes we eat, sometimes we don’t. People don’t know the reality inside. They only see from the outside and assume we are well-off. …I try to stay cheerful, but no one knows the pain I carry inside.”

Like this woman, many participants in the study had to make impossible choices to meet basic needs, alternately foregoing food, medicine, or improved housing. One woman reported, “Food is a big concern. Though I’ve married off one daughter, I still have five more to care for. Sometimes we eat, sometimes we don’t. …I go without eating just to raise my children well, to keep them happy.” Another woman had been looking toward building a permanent structure to replace their mud hut, but her husband’s intermittent illness made it impossible to afford. “We can’t seem to manage,” she lamented. “We had bricks—6,000 bricks to be precise [for building a house]. Back then, I joined a savings scheme on installments. We used money from my husband’s work, and we were doing okay. We had stored the 6,000 bricks, but later, we had to sell all of them.”

#### Debt

As noted above, over ninety percent of women in the study had a current loan, and often more than one. Women-focused lending programs in this area require frequent repayment installments, which were both stressful and unrealistic for many. “On Tuesdays, I have to pay 1,600 takas to UDB,” explained a participant. “On Thursdays, 600 takas to Baish Mitali. I have to pay 2,200 takas per month to BRAC. …In another place called Palli Shree, I have to pay 500 takas per week.” She explained that she paid from her husband’s daily wages when possible, and otherwise borrowed from high-interest formal or informal lenders around her neighborhood. This cycle of debt and borrowing was deeply anxiety-provoking for many women in the study. “As soon as I wake up, the loan collectors come to me,” said one woman. “They always come to the woman of the house, and the loans are taken in the woman’s name. I am always in a state of worry about this,” she reported. Other women talked about the humiliation of being unable to repay loan installments. When asked about the biggest cause of stress in her life, one participant exclaimed, “Debt, of course! I survive on loans. I have a loan of 40,000 taka. Every week, I have to pay 700 taka. When I can’t manage the installment, I borrow from moneylenders to pay it off. …If I can’t pay an installment, the collection agents come and sit at my house, waiting. Then the neighbors start talking badly, saying, ‘She borrowed money but now can’t pay it back.’ That’s embarrassing.” Both these women, and many others, were locked into cycles of debt where they were using one loan to pay off another, while accruing ever more interest on their borrowed money.

#### Health problems

Many women reported health problems among their family members that jeopardized income or consumed savings. Women struggled with husbands’ or elderly in-laws’ illnesses and often found themselves sandwiched between care for children and care for ill adults in their households. One woman whose husband had died in the past year said, “Among us, I was the only healthy one, so I did all the work—both at home and outside.” As her husband’s health deteriorated, they borrowed money first from her father-in-law and eventually from her father. This resulted in both sets of parents eventually cutting them off. Her father-in-law told her husband, “You must earn your own living. I raised you; now should I support you and your children too?” After an altercation with her own father, they became estranged and had never spoken again. “My father was upset that his daughter had taken so much money from him,” she explained, but the family was destitute. “Later, when my eldest son was bitten by a dog, we couldn’t afford his treatment. At that time, a rabies vaccine cost about 600–700 taka, and we didn’t have that money. …Life has been full of struggles.” Basic supplies like nutritional supplements to address deficiencies from food insecurity were often impossible to afford as well. “I have a severe calcium deficiency and lack of iron in my body. Because of this, I get sores that ooze. …[The doctor] told me to take [supplements]. But due to a lack of money, I can’t take them regularly. I get them whenever I have the money, but I can’t complete the full dose.” These medical shortfalls further entrenched women’s poverty in many ways: by reducing their ability to work, making them more susceptible to additional health problems, increasing their debt, endangering family relationships that might otherwise be a source of future support, and consuming assets that could otherwise generate income.

Health problems among family members were another source of strain, especially in the context of extreme poverty. “[My son] works as a day laborer, doing people’s [farm] work, but now there’s no work. Also, the baby has had diarrhea since yesterday. I took him to the doctor, and they gave me medicine for 600 taka. I bought medicine for 300 taka, but I told them I had no more money, so they reduced the dosage and said I could come back later,” explained one woman. She could not afford soap to wash the soiled sheets from her son’s diarrhea. “He’s having diarrhea six or seven times at night. All the blankets and sheets are ruined. I have to wash everything, but I don’t have money to buy soap. I need soap to wash everything. What else can I say?”

#### Land scarcity

In this agricultural region, struggles over land ownership and inheritance were common, with 37 out of 60 participants discussing land-related challenges. Housing insecurity was a significant source of distress for study participants who did not own the land on which they lived. When asked if she felt her worries were more or less than her peers, one woman replied, “Since I have nothing, I will obviously worry more. …I don’t even have my own land to live on. I need to secure a home, acquire farming land, build a house. That’s why my worries are greater. …Other people already have homes, farming land, so they don’t have to worry about these things.”

Land conflicts were a major source of family feuds. Typically, senior men divided up their land holdings among their sons (and sometimes daughters) either at the children’s marriage or at death. But women reported multiple instances of other family members tricking them out of their inheritance or refusing to vacate land, which contributed to stress by compromising housing insecurity and promoting family conflict. “Not being able to build a proper house is one of my biggest worries,” reported one woman. “And after marriage, the biggest stress that constantly lingers in my mind is my husband’s paternal aunt. She is living on our land and refuses to leave. We want to build our house there—we have already deposited 50–60 thousand takas for bricks two years ago. But we can’t start construction until she moves out.” The aunt’s claim was endangering her husband’s rightful inheritance. “[My father-in-law has] just two *shatak* (about 870 ft^2^) of land. Out of that, my paternal aunt-in-law is claiming half a shatak. So, how much is left for my father-in-law? Just one and a half shatak. And within that, my brother-in-law also has a share. So, when you look at it, my husband is left with nothing. Absolutely nothing.” Intra-family deceit was also common. One woman told how her older brothers-in-law persuaded their sick father to sign documents that cut her husband entirely out of the inheritance. “My husband and his brother didn’t get any land from my father-in-law. The other two brothers took it through deceit. They got my father-in-law to sign over the land and house when he was unwell, saying it was for his treatment. That’s how they took it.” These land grabs were particularly contentious because land was so scarce and financial insecurity so great.

#### Worries about children’s future

Women universally desired a better future for their children, and they were often deeply distressed by the struggle of trying to improve their life chances with so few resources at hand. One woman said, “Since I didn’t get an education, I at least understand the importance of making sure my daughter does. No matter what it takes, I will work every day if I have to, just to educate my daughter. Because wherever I go, I face struggles due to my lack of education. Now I truly understand the value of it.” Another remarked, “Even if I can’t achieve much for myself, I’m trying to ensure my daughter gets an education and becomes someone in life.”

Though women had high hopes for their children’s futures, they also faced financial and social pressure to participate in practices like dowry and early marriage for their daughters, and this added layers of worry about girl children in particular. One said:

My daughters are growing up. And not just one—I have two daughters. I have to think about their future. What if they end up getting married young like I did? And if their fate turns out as bad as mine, how will that be? Bangladesh has changed now; girls are moving ahead even more than boys. I have big hopes that my daughters will progress. I don’t want them to end up like me. I want them to have financial security.

As this comment illustrates, women’s concerns for their children’s future were intertwined with the other concerns we have detailed in this section, such as the high cost of medical interventions, housing insecurity, and (as we will discuss further below) dowry and child marriage. Ultimately, women fervently hoped to secure a better future for their children, and this was both a key motivator and source of strain in their everyday lives due to poverty constraints.

### Theme 2: Patriarchy and its consequences

Patriarchy—its norms, practices, and the deprivation and social marginalization often associated with them—infiltrated every aspect of women’s lives. Every woman in the study described challenges directly related to patriarchal norms. These include patrilocality, marital conflict, child marriage, mobility restriction (sometimes motivated by *purdah*), and dowry disputes. We detail each of these concerns below.

#### Patrilocality

All women had moved to their husbands’ villages, and over 50 percent had moved directlyinto their in-laws’ households, after marriage. These cohabitation arrangements tended to be unstable, however, and only five women were still cohabitating and sharing food with their in-laws at the time we interviewed them.

Patrilocal marriage separates women from childhood social and family connections, leaving their social lives tightly circumscribed (Hruschka et al., 2022; Jeffery, 1979; Wiser & Wiser, 1971). Women looked back wistfully at childhood as a time when they had enjoyed both freedom and sociality. “Before marriage, I had friends, but now we are all in different places. They are married and living in one place, and I am in another,” said one young woman. For many women, patrilocal marriage created isolation and emotional dependence on their husbands and in-laws, a theme well documented in the literature (Jeffery, 1979; Wiser & Wiser, 1971). Another participant explained, “Now, after marriage, I don’t share [personal problems] with anyone. But back in school, I had a friend I used to share with… you know, school life stories. Now it’s married life. [My friend] is also married, and we don’t meet anymore. So, I don’t share things now.” When asked about whether she ever felt lonely, this participant explained, “Well, you see, I’ve come here because of my husband. He is the closest person to me now [post-marriage]. When he scolds me a bit or says something hurtful, that’s when I feel [lonely].”

Some women did eventually develop friendships in their affinal communities which could serve as important sources of social support. But as dependent members of affinal households, they experienced heavy constraints on their ability to form close relationships with other women. Participants described hesitating to share personal problems with people outside the household, since gossip was common. One woman described an incident five year prior when she had discussed a family conflict with a neigbhor and endured beatings from her husband and in-laws after it got back to them. “Now, I don’t go to anyone,” she said. “Who can I build a close relationship with? Whoever I get close to, it leads to problems.” To avoid this risk, some chose friends who weren’t part of their immediate communities. For instance, one woman had befriended her younger brother’s wife, whom she visited with whenever she saw her parents. “I feel good talking to her, and she tells me that if I do things this way, then that will happen, so why worry. …I don’t talk to anyone else about [my worries]. I fear that if I tell someone else, they might think badly of me or misunderstand. I don’t share these things with any neighbors or anyone else.”

#### Marital conflict

The single most important relationship of women’s adult lives was with their husbands. Husbands could be a major source of emotional support for women, but a lack of emotional intimacy, addiction, and violence were common. About one-third of the women characterized their husbands as “not good”—not working adequately to support their wives and children, gambling, using substances, or being physically or sexually abusive.

Seventeen out of 60 women reported their husbands experiencing addiction. Though Bangladesh is technically a dry country, bootlegged alcohol and cannabis are sold illegally, and most pharmaceuticals are available without a prescription, leading to dependence on addictive medications like opioids and benzodiazepines. Addiction among men was endemic in some villages. “This area is not a good place at all—people here take drugs. Even [my husband] takes them,” reported one woman. “The people in this area are excessive—almost everyone is addicted.”

Physical abuse was common among women whose husbands were “bad.” “If I delayed any work, he would hit me,” said one participant. “If the rice took too long to cook, he would slap me. If there was a disturbance at home, he would ask, ‘Why is the cooking taking so long?’ I might have said there was no firewood, or the baby was crying. Sometimes, if I wasn’t feeling well and couldn’t cook, he would beat me for that too.”

Abusive marital relationships relationship permeated every aspect of women’s daily lives and contributed to distress through both the direct effects of violence and the indirect effects of lack of care on household financial stability. One woman explained, “My husband is not a good man. He doesn’t want to contribute to our children’s education expenses. He only thinks about feeding himself. Now I have to take responsibility for running the household by myself. He only cares about his own needs.”

#### Child marriage

Child marriage was universal in our sample; all participants reported being married between the ages of 14 and 16. They described this as a source of stress because it foreshortened their educational aspirations and ensured they would enter their affinal households as the most junior member. Though illegal, child marriage is easily accomplished in rural Bangladesh through various subterfuges, such as forging the bride’s age on ID cards or birth certificates. There was a widespread belief in the study setting that unmarried girls present a social and reputational risk to their parents, especially after menarche. As one participant explained, “In our community, they don’t keep an unmarried girl once she’s considered *seyanna* [literally, ‘clever, grown-up,’ referring idiomatically to menarche]. As soon as she grows up a little, they arrange her marriage. …When I got married, I was 14 years old.” The fear of rumor-mongering drove many parents to arrange their daughters’ marriage early, before they could get involved in romantic liaisons that might endanger their reputations. Even women who opposed early marriage experienced social pressure to marry their daughters off. “Many people keep talking about my daughter’s marriage. They think she’s 14, and according to our tradition, a girl should marry at 18, but many are getting their daughters married even at 16, when they find a good match,” explained one woman. She didn’t believe in early marriage, however, and recognized the threats it could create to her daughter’s wellbeing. “It really bothers me when people tell me to marry my daughter off.”

Early marriage had ripple effects on women’s entire lives. Though it would theoretically be possible for a girl to continue studying after marriage, in practice this did not happen, since she was expected to take over household responsibilities in her in-laws’ home, and in-laws were not expected to pay for further studies. “Of course, I wanted to study. Who doesn’t want to study and become somebody?” asked one participant who studied only until the sixth class. Poignantly, one woman recalled a turning point where she might have had a rewarding career if circumstances had been different. “Even now, I dream of studying. I dream that I’m taking exams or studying. I still dream about it. …I wanted to teach young children. There was a primary school near our house. …There were little children, but no female teachers —only male teachers. They said that if I completed my matriculation, they would hire a female teacher. That was my dream.”

Beyond a lack of education, early marriage had other impacts that set women up for a lifetime of deprivation, such as having children early and limiting the possibility of employment. In the words of one woman, early marriage made women’s lives feel “small”: “Sometimes I feel very small… I feel sad. I often think that if I had been allowed to study, I could have stood on my own feet… I wouldn’t have had to struggle financially. I could have met my own needs. Thinking about this makes me feel really bad. Whenever we face financial trouble, I remember this and feel even worse.” Additionally, early marriage magnifies power differentials between young brides and their in-laws, since a very young bride is less likely to be able to advocate for herself during family disputes or abuse.

#### Mobility restriction

Most women reported experiencing some type of family-level restriction on their freedom of movement outside the home; 34 specifically reported that they had to ask for permission from their husbands, in-laws, or adult sons before going out. When they did go out in public, many women were required to take along a husband, child, or sister-in-law for small errands such as picking up cooking supplies or dropping children at school.

Women drew on varying logics to explain the purpose and value of mobility restriction. Many Muslim women explained mobility restriction in terms of *purdah*. One woman explained, “There is an issue due to purdah. For example, being around men, talking to them, or even walking in their presence can be problematic. That’s why I prefer not to go out alone very often. According to Islamic teachings, it is forbidden to speak to men, and interacting with them is considered sinful.” Other women explained mobility restriction in terms of concerns about personal or family reputation. As one woman explained, her husband was suspicious of other men, and this partially motivated his control of her movements. “If I talk to a man, he assumes there’s something going on. That’s why I always take his permission before going anywhere.” In-laws often intentionally limited women’s interactions with people outside the household out of fears of gossip or concerns that their daughters-in-law might reveal abuses going on within the home. “My mother-in-law doesn’t like me visiting neighbors. She thinks that if I talk to people, I might gossip about her or my sister-in-law. She suspects that I might share household matters with others. Because of this, I used to visit before, but now I don’t go much. I just do my household chores and sit with my children.”

For some women, mobility restriction was unbothersome, but for others, it created tension. One woman explained, “I have no freedom, sister….[My husband] says, ‘Why do you need to go?’ Even if I go to my sister’s house, he questions why I’m going!” Even when husbands granted permission for women to travel, the permission-taking process might generate delays that limited women’s ability to go. One woman, who lived very close to her natal family, reported visiting them only rarely “because I have to take permission first. By the time I get permission, seven or eight months just pass by.” Although mobile communication made staying in touch easier for some women, many didn’t own a mobile phone and had to ask a male relative if they wanted to make a phone call.

#### Dowry disputes

Dowry was a source of tension in women’s lives because it created financial obligations between affinal and paternal families that often extended many years after marriage, and in many cases, kept women financially dependent in their affinal households. Forty out of the 60 participants reported an existing dowry-related conflict within their marital families. Women often felt that they should have some claim to their dowry, but it was almost invariably controlled by their husband or in-laws and often spent without their consent. “When I went to my in-laws’ house, I was given nothing. Since [my mother-in-law] raised her son, she claimed that money was rightfully hers,” explained one woman. Another said, “I couldn’t even keep a goat for myself—everything belonged to them. Even the room we stayed in wasn’t truly ours.

Whenever there was a fight, they’d tell me to leave and lock the door. My father-in-law used to say, ‘Get out! What did you even bring from your father’s house?’ Even after bringing so much dowry, I still had to hear these insults.” These incidents could be emotionally as well as financially devastating. One woman whose mother-in-law took her costly gold earrings said, “Since then, I have stopped dreaming. When I told my husband, he said, ‘Don’t have expectations from people; I will get you jewelry myself.’ Now I don’t dream anymore.”

#### Conflicts

over dowry often went on for many years after marriage, with in-laws harassing a woman and her family for outstanding dowry installments or treating women’s parents as a “bank” when needs arose. This could lead to major family conflict, which compounded women’s social and emotional isolation. Even after her dowry payments were complete, one woman’s in-laws continued to demand resources from her parents. “They thought that since I was an only daughter, my parents would give me a lot of things. I had already brought dowry, but still, they wanted more! If they wanted to build a new house, they made me go and bring bamboo [from my parents].” Another woman explained that her father became unable to give a bicycle he had promised when all his crops failed one season. But her in-laws would not relent. “I tried to explain to my husband, ‘How will my father give the money? He has no means now.’ …Sometimes, hearing people’s words [gossip], [my husband] would slap me once or twice. I would cry, then stay silent.” The nagging, gossip, and physical and verbal abuse escalated until her father took a high-interest loan to buy the bicycle, hoping to appease the in-laws and make his daughter’s life more tolerable. Yet even after receiving the bicycle, the in-laws continued to harass her father for money, resulting in yet another high-interest loan on his part. Though the dowry was finally paid off, the woman had five children in six years and her husband couldn’t work steadily because of a respiratory illness. Out of desperation, she continued going to her father for money, but this eventually resulted in permanent alienation between father and daughter.

In a few instances, there was no dowry, and this too generated long-lasting conflict. One woman reported that the lack of dowry created poor relationships with her in-laws. “Actually, ever since I got married, I haven’t had a good relationship with my sister-in-law. I eloped. If my marriage with her husband’s younger brother had been arranged properly, they would’ve received money or dowry. But because we eloped, they didn’t get a share of any dowry. She taunts me about this.” Another woman was deeply stressed about her own recently-married daughter’s fate because they had not been able to pay dowry. The new in-laws often dropped hints about the lack of dowry or outright derided her daughter. “Sometimes I even cry, thinking about how these comments might hurt my daughter. If only I had the means, [her parents-in-law] wouldn’t be able to say such things. Her other in-laws also say things like, ‘What are you bringing from your father’s house?’.” Women were generally stressed by dowry—for both themselves and their daughters—but faced lasting social consequences if they opted out.

### Sources of resilience

Amidst this incredibly challenging set of circumstances, women named a few resources that helped them cope with tension.

As noted above, a “good” husband was considered a very valuable asset in women’s lives, and about two-thirds of the participants described at least some parts of their marital relationships positively. For some women, “good” husbands were confidants, companions, and sources of meaningful financial and emotional support. “I have a very good relationship with my husband. We share everything, so I don’t feel that much hardship,” said one woman. Even though they struggled with basic needs, she did not feel alone. “He loves me a lot,” she went on. “He also helps with work many times.” A good husband treated both a woman and her family of origin with respect. “*Alhamudillah* [thanks be to God], it’s quite good,” said one. “He has never laid a hand on me until now. He has never scolded or insulted my parents in a bad way either. Overall, things are quite good, *alhamdulillah*. It’s more like a friendship—we share everything.”

Though in-laws often discouraged women from in sharing their troubles outside the home, many women had at least one friend on whom they could rely in times of stress, and this was another resource that helped women cope with tension. One participant said, “She can tell by looking at me that I’m worried about something. Then she comforts me, saying, ‘Don’t worry, Allah is watching. One day you’ll find happiness. Why stress so much?’ …I let out a bit of what’s on my mind and feel better after talking to her.” Women described feeling “lighter” after sharing troubles with others. “I would feel lighter, and there were 14 others besides me and also two madams,” said one woman, referring to the other women in her intervention group. “Getting advice from so many people would be helpful, that’s why I shared [my troubles]” said one.

Finally, many women received support from their natal families (though, as noted above, this was not always possible due to mobility restrictions and financial strain). Some women only visited their natal families rarely, but stayed in close touch by phone. One woman said, “I go there once a year. My parents come here too. Right now, my mother and brother are here. We also talk over video calls, so we stay in regular contact. They’ve been here for 14 days and will leave tomorrow. If I need money, they help.” A few women visited their parents frequently, although this was rare. One woman explained, “Whenever I feel down, I go to my parents’ house. My mother cooks food for me and keeps it in the fridge.” This woman’s sister also supported her both financially and emotionally. “A few days ago, I was facing severe financial problems, so one of my sisters gave me 8,000 taka. Out of that, I used 3,000 to pay off a [loan] installment. I didn’t have any work then, and you know, you can’t survive just by harvesting potatoes occasionally. I am very close to this sister. We share everything, all our joys and sorrows. We talk over the phone every day.” Natal families often willingly sacrificed to provide for their daughters or siblings, even though it was not their social obligation to do so. “I ask my younger sister because she works in Dhaka, and she doesn’t refuse me. Whatever I ask for, she gives if she can,” said one participant.

Each of these relationships reduced women’s distress because they provided an emotional connection outside their affinal families, gave women a sense that they were not handling their problems alone, and connected women to essential emotional and financial resources.

## Discussion

This study was built around the idea that talking to people about distress can reveal features of the “moral and social order” of their everyday lives (Kleinman 1988: 31). At the broadest level, our thematic analysis of interviews with rural Bangladeshi women experiencing likely depression identified poverty and patriarchy as the two key features of the moral and social order that underpinned many of their stressors. For instance, aspects of poverty like dowry disputes, land scarcity, and debt were deeply contentious *precisely because* resources were too limited to meet every family member’s needs. Likewise, women were in positions of less power to negotiate within their families *precisely because* of patriarchal practices such as child marriage, dowry, and patrilocal settlement. For women in our study, key sources of distress were debt, basic resource insecurity, health problems, land scarcity, worries about children’s futures, marital conflict, social isolation created by patrilocality and mobility restriction, child marriage, and dowry conflict. Women found spaces of support and resilience among their social communities but still contended with daily struggles of both an emotional and a practical nature.

Women’s struggles with poverty and patriarchy in this study are consistent with global development and demographic literatures, which demonstrate that women in many parts of Bangladesh face basic needs challenges such as lack of education, early marriage, financial precarity, treatable health problems, and vulnerability to climate crises (Islam & Sayeed, 2024). The Global Gender Gap Index, a composite measure of gender equality across economic, educational, political, and health realms, ranked Bangladesh 99th out of 145 countries in 2024 (World Population Review, 2024). The centrality of poverty and resource scarcity in this study also echoes a prior systematic literature review on “tension” in South Asia, which found that across 122 studies, poverty was the most common cause for “tension” (Weaver & Karasz, 2022). Though women in this study aspired to a better life, they were entrenched in poverty because they were unable to accumulate assets such as land and livestock (Davis, 2011). The lives of the women in this study demonstrate how “durable inequality” persists in Bangladesh, even though women have keen aspirations for a better life (Bhattacharjee & Talukder, 2021).

Many of our findings are consistent with the 20th century ethnographic record from Bangladesh. As noted in the Introduction, dowry, patrilocality, and purdah were common threads in the ethnographic literature dating back to the 1920s (Wadley, 1994; White, 1992; Wiser & Wiser, 1930). In our work, control of women’s movement, behavior, and sexuality continued in line with earlier ethnographies as a way to protect the family’s social reputation (Liddle & Joshi, 1989; Mandelbaum, 1988). This was reflected in our results, for instance, when parents married their daughters off early to avoid the possibility of socially damaging love affairs, or when in-laws discouraged women from forming friendships with neighbors out of fears of gossip. Mobility restriction was not, however, always conceptually tied to religious practice of purdah and was often instead described in secular terms as a practical measure to protect women’s safety or the family’s reputation.

Another important point of continuity with prior ethnographic work was family structure. Though the normative arrangement was, and still is, a multi-generational cohabitating joint family (Wiser & Wiser, 1971), the literature also reflects a common pattern of joint family fission in very poor households (Lieten, 1993; Van Schendel, 1982; Wadley, 1994). All women in our study had moved to their husbands’ and in-laws’ villages after marriage, but many cohabitating joint families had dissolved over time because of land disputes, general resource scarcity, or intra-family conflicts that made families want to separate.

Though there was significant overlap between women’s experience in our study and the ethnographic literature, we also noted some important differences. Previous work describes women’s natal families as having a very circumscribed relationship with their daughters after marriage (Wadley, 1994; White, 1992). Close ties with the natal family remained but were considered “an important back-up” providing temporary respite from a difficult marriage (White, 1992; Wiser & Wiser, 1971), rather than an everyday source of support. Many women in our study, by contrast, reported ongoing financial and emotional support from their natal families, sometimes even many years after marriage. We suspect that this finding is partially because women in the study are exceedingly poor and may rely on their natal families out of sheer necessity. It could also reflect the advent of mobile communication in this region, which made it easier to stay in touch with natal families.

One potentially “new” problem not reflected in the prior literature was husbands’ misuse of prescription medications such as opioids and benzodiazepines. There are reports of bootlegged alcohol and cannabis use among men in North India (Wadley, 1994; Wiser & Wiser, 1971), but no ethnographic record in Bangladesh. The steep rise of prescription drug misuse is only recently appearing in the medical literature from Bangladesh, likely a result of the global opioid crisis and the very loose regulation of pharmaceuticals across South Asia (Malla et al., 2015).

Another “new” stressor reported by many women in our study—and documented in recent ethnographic work—is the burden of debt. Bangladesh’s women-centered development programs have elevated women as important drivers of economic change (Islam & Sayeed, 2024; Nazneen et al., 2011), as in the Grameen Bank, a model of microfinance developed in rural Bangladesh and popularized around the world (Yunus, 2007). Over ninety percent of women in our study were current participants in similar programs, which on the one hand opened up new avenues for loans, but on the other hand, focused the burden of debt and its repayment solely on them. Karim’s (2011) and Murshid’s (2018) work on microfinance demonstrates negative consequences for Bangladeshi women borrowers, including becoming entrenched in debt, intra-family domestic violence, and public shaming and stigma. Our findings echo this work and illustrate how development projects can produce what Chowdhury refers to as the intertwined “predicament of women’s oppression and liberation” (Chowdhury, 2018: 47), where lofty claims about women’s empowerment reflect “elite discourses rather than [the experiences] of the majority of Bangladeshi women” (Nazneen et al., 2011: 7).

Overall, our discussions with women about distress illuminated the “moral and social order” (Kleinman 1988: 31) of patriarchy and poverty in which they lived. These forces profoundly limited their children’s and their own life chances. Our results point to significant continuity with concerns documented in 20th -century ethnographies, along with some newer concerns stemming from development projects and the pharmaceutical industry.

## Conclusions

This study examined the narratives of women with likely depression in rural northern Bangladesh to understand the fault lines in their moral and social worlds (Kleinman, 1988). Social institutions reflected in the ethnographic record, like dowry, patrilocality, and mobility restriction, were still key features of life for women in the study and were significant sources of distress in women’s lives. On top of these “classic” stressors, newer stressors also surfaced from women’s narratives, including debt to microlending programs and husbands’ addictions to opiates and benzodiazepines. Women articulated an awareness of emerging opportunities for girls and women in rural Bangladesh that did not exist a generation ago; things like education, marrying later, having smaller families, and working in a job to have financial independence. They hoped for their children to benefit from these new opportunities, but their aspirations were often thwarted by ongoing poverty and patriarchal norms that limited women’s and girls’ life chances.

The next generation of rural Bangladeshi women may have more opportunities for empowerment than the women in our study. Yet, Chowdhury (2018) argues that “social and cultural mobility remain elusive for most Bangladeshi women” (Chowdhury, 2018: 62). For women in our study, the consequences of ongoing patriarchy and poverty were profoundly important social problems made visible by their discussions of everyday stresses. The continuity of women’s experiences with much of the prior ethnographic record suggests that the promise of social and cultural mobility for Bangladeshi women falls short of women’s lived realities.

## Figures and Tables

**Table 1:** Participant demographic characteristics (n = 60) at baseline.

**Age (mean, range)**	30.0 (23.5–36.5)
**Age at marriage (mean, range)**	15 (14–16)
**Education in years (mean, range)**	6.5 (2–12)
**Number of children (mean, range)**	1.77 (0–4)
**Cohabitates with in-laws**	32 (53%)
**Religion**	
Christian	1 (1.7%)
Hindu	14 (23.3%)
Muslim	45 (75.0%)
**Marital status**	
Currently married	55 (91.7%)
Separated	2 (3.3%)
Widowed	3 (5.0%)
**Monthly household income (tk, mean, range)**	11,625 (9,024–14,249)
<=5000	2 (3.3%)
5001–10000	16 (26.7%)
>=10,001–15000	42 (70%)
**PHQ-9 Score (mean±SD)**	12.8 (±2.4)
Moderate depression	49 (81.7%)
Moderately severe depression	10 (16.7%)
Severe depression	1 (1.7%)
**Current Loan**	
Yes	55 (91.7%)
No	5 (8.3%)

## Data Availability

Due to the sensitive nature of the data used in this study and the risk of loss of confidentiality, the data are not available in a public repository. De-identified data are, however, available from the authors upon reasonable request.
